# RhoA Regulation of Cardiomyocyte Differentiation

**DOI:** 10.1155/2013/491546

**Published:** 2013-06-27

**Authors:** Mari Kaarbø, Denis I. Crane, Wayne G. Murrell

**Affiliations:** ^1^Department of Microbiology, University of Oslo, Oslo University Hospital, Rikshospitalet, 0454 Oslo, Norway; ^2^Cell Biology, Eskitis Institute for Cell and Molecular Therapies, and School of Biomolecular and Biomedical Science, Griffith University, Nathan, QD 4111, Australia; ^3^Vilhelm Magnus Laboratory for Neurosurgical Research, Institute for Surgical Research, Oslo University Hospital, Rikshospitalet, 0454 Oslo, Norway

## Abstract

Earlier findings from our laboratory implicated RhoA in heart developmental processes. To investigate factors that potentially regulate RhoA expression, RhoA gene organisation and promoter activity were analysed. Comparative analysis indicated strict conservation of both gene organisation and coding sequence of the chick, mouse, and human RhoA genes. Bioinformatics analysis of the derived promoter region of mouse RhoA identified putative consensus sequence binding sites for several transcription factors involved in heart formation and organogenesis generally. Using luciferase reporter assays, RhoA promoter activity was shown to increase in mouse-derived P19CL6 cells that were induced to differentiate into cardiomyocytes. Overexpression of a dominant negative mutant of mouse RhoA (mRhoAN19) blocked this cardiomyocyte differentiation of P19CL6 cells and led to the accumulation of the cardiac transcription factors SRF and GATA4 and the early cardiac marker cardiac **α**-actin. Taken together, these findings indicate a fundamental role for RhoA in the differentiation of cardiomyocytes.

## 1. Introduction

One of the challenges of development research is to identify factors that control the differentiation of specific cell types such as cardiomyocytes during the various stages of early heart development. Earlier work conducted in our laboratory, employing differential display and *in situ* hybridisation, indicated that RhoA was upregulated in the stages of early heart development [1]. Specifically, immunocytochemical analysis revealed marked upregulation of RhoA in heart primordial regions (stages 6–8) and disruption of RhoA expression *in vivo*, using small interfering RNA injected into lateral plate mesoderm of early chick embryos, resulted in cardia bifida and abnormal head development. These findings thus implicated RhoA as an important factor for normal development of heart and head formation [1]. 

RhoA is a small GTPase that acts as a molecular switch to control a variety of signal transduction pathways in eukaryotes. RhoA's first characterised function was in the regulation of the actin cytoskeleton, but it has now been implicated in a variety of other functions including regulation of gene transcription and of processes affecting morphology. Magie and colleagues reported that loss of the RhoA ortholog *Rho1* in *Drosophila* results in severe defects in morphogenetic processes such as defective head involution and imperfect dorsal closure in embryos [2]. In *Xenopus*, *XRhoA* has been suggested to be the first intracellular signalling molecule implicated in head formation [3].

However, other evidence suggesting a specific role for RhoA in the molecular pathways of early cardiogenesis is also emerging. For example, Wei and coworkers reported an essential role in vertebrate embryonic organogenesis for Rho associated kinases (Rho kinases), direct downstream effectors of RhoA. In its active GTP state, RhoA activates Rho kinases which then phosphorylate downstream targets. Rho kinases thus mediate many *in vivo* functions of RhoA. Importantly, inhibition of these Rho kinases in early chick embryos blocked migration and fusion of the bilateral heart primordia and induced expression of cardiac *α*-actin, SRF, and GATA4 mRNA levels in stage 8 embryos [4]. Wei and coworkers also demonstrated that cardiomyocyte-specific inhibition of all the Rho GTPases resulted in disruption of cardiac morphogenesis, including cardiac looping and chamber maturation, suggesting a critical role for Rho GTPases in murine cardiac development [5]. In this paper, we show that specific inhibition of RhoA inhibits cardiomyocyte differentiation.

Significant progress has been made in defining pathways involved in the formation of the heart of higher vertebrates. However, little is known about the genetic program that determines the differentiation of cardiomyocytes from their precursor cells. In order to investigate the RhoA regulation process of cardiogenesis at a cellular level, the murine P19CL6 cell line was developed. This cell line is a clonal derivative isolated from pluripotent P19 embryonic carcinoma cells. Compared to its parental cell line (P19 cells), P19CL6 cells differentiate efficiently into cardiomyocytes when treated with 1% dimethyl sulfoxide (DMSO) [6].

In the study presented here, the comparative organisation of the chick, mouse, and human RhoA genes was determined to allow identification of factors that regulate RhoA expression in the developing heart. The majority of consensus transcription factor binding sites identified in the putative RhoA promoter have previously been implicated in either heart development or organogenesis. We report that the promoter activity of RhoA is increased when P19CL6 cells are induced to differentiate into cardiomyocytes and that over-expression of a double negative mutant of mouse RhoA (mRhoAN19) blocked the cardiac differentiation of induced P19CL6 cells and led to an accumulation of the cardiac transcription factors SRF and GATA4 and the cardiac marker cardiac *α*-actin. These results are compatible with an important role for RhoA in early cardiogenesis.

## 2. Experimental Procedures

### 2.1. PCR of Chick Genomic DNA

Chick genomic DNA was extracted from six-day chick embryos using the following procedure: embryos were added to one volume of lysis buffer (50 mM Tris-HCl pH 8, 20 mM EDTA, 2% SDS), mixed with a 1/20 volume of 10 mg/mL proteinase K, and digested overnight at 60°C. The solution was then chilled on ice for 10 min before being mixed with a 1/3 volume of saturated NaCl and incubated for a further 5 min on ice. The solution was centrifuged at 14 000 ×g for 20 min at room temperature. The supernatant was transferred to a fresh tube and mixed with one volume of isopropanol, and the DNA was pelleted by centrifugation at 14 000 ×g for 10 min at room temperature, washed with 70% ethanol, and repelleted by centrifugation at 14 000 ×g for 5 min at room temperature. The gDNA pellet was dried and resuspended in TE (10 mM Tris-HCl, pH 8.0, 1 mM EDTA). This genomic DNA was then used as template for PCR using Taq polymerase (Invitrogen) and primers (see Table 1) spanning the putative intron sites of the chick RhoA gene identified by comparative analysis of RhoA cDNA and gDNA sequences. 

### 2.2. Cloning of the Promoter Region of RhoA

The putative promoter region of mouse RhoA and sequences corresponding to transcription factor binding sites in this region were identified by computer analyses using the Gene2Promoter, MatInspector, and PromoterInspector computer tools (http://www.genomatix.de). Mouse genomic DNA, extracted from P19CL6 cells using the same procedure as described above, was used as template to PCR amplify the two different length fragments of the putative promoter region of RhoA utilising the GC rich system (Promega) and the primers described in Table 1. The two products were digested with *BglII* and independently cloned upstream of the firefly luciferase coding sequence, at the *BamHI *site of pGL3-Basic vector. 

### 2.3. Cell Culture and Differentiation

P19CL6 cells were cultured as described previously [6] but with some modifications. Briefly, cells were cultured in a growth medium (GM) consisting of minimal essential medium (Invitrogen) supplemented with 10% fetal bovine serum (Invitrogen), penicillin (100 U/mL), and streptomycin (100 *μ*g/mL). To induce differentiation into a cardiomyocyte phenotype under adherent conditions, cells were plated at a density of 1.67 × 10^5^ cells/2 mL or 3 × 10^4^ cells/0.5 mL DM into 6-well and 24-well plates, respectively, in differentiation medium (DM: GM containing 1% dimethyl sulfoxide (DMSO)). The medium was changed every second day, with the first day of DMSO treatment designated differentiation day 0 (D_0_).

### 2.4. Cell Transfections and Reporter Assays

Eleven days prior to transfection, P19CL6 cells were plated in 48-well plates in 0.25 mL GM or DM at a density of 19 000 cells/well. Three independent transfections were performed in triplicate for all samples. For each transfection, cells were incubated with Lipofectamine 2000 (Invitrogen) containing 800 ng of RhoA expressing test plasmid (cloned into pGL3-Basic: express firefly luciferase) and 80 ng of coreporter plasmid (pRL-TK: expresses* Renilla *luciferase) (Invitrogen), according to the manufacturer's instruction. The following day the cells were assayed for expression of luciferase activity using a Victor 1420 multilevel counter (Wallac, Turku, Finland) and the Dual Luciferase Assay system (Promega), as per the manufacturer's instructions. Assay background was measured by including cells incubated with pGL3-Basic lacking insert and Lipofectamine 2000 alone. 

### 2.5. RNA Preparation, cDNA Synthesis, and Cloning of RhoA Constructs

Total RNA was extracted from cultured P19CL6 cells using a combined method based on the RNA extraction protocols of Chomczynski and Sacchi (1987) and Zimmermann and Schultz (1994), described previously [1]. Superscript III reverse transcriptase (Invitrogen) was used as per the manufacturer's instructions to generate first-strand cDNA. To generate the dominant negative form of the mRhoA protein, a 5′ primer incorporating sequence flanking the ATG start codon of mouse RhoA cDNA and the mutation encoding the dominant negative mutated form of the protein (mRhoAN19) was used in combination with a 3′ primer corresponding to sequence near the 3′-end of the mRhoA ORF (see Table 1). PCR was undertaken using the Expand high fidelity system (Roche) and the product TA-cloned into the pTARGET expression vector (Promega) and confirmed by direct sequencing.

## 3. Generation of Stably Transfected Cells

Two days before transfection, 1.7 × 10^5^ P19CL6 cells were plated out in 2 mL GM without antibiotics in 6-well plates. The cells were transfected with the pTARGET-based RhoA expression vectors, or vector alone, using Lipofectamine 2000. Stable clonal transformants were selected with 600 *μ*g/mL Neomycin (G-418, Invitrogen). Incorporation of the plasmids, with incorporated RhoA sequence, into the genome of the different P19CL6 cell lines was confirmed by genomic PCR and sequencing. 

## 4. Immunocytochemistry

For immunocytochemical detection of cTnI, cells were fixed in 4% paraformaldehyde in PBS, pH 7.4 at room temperature for 10 min, and washed three times for 5 min in PBS. Endogenous peroxidase was then quenched by incubation in 0.3% H_2_O_2_ for 30 min. The fixed cells were washed again twice in PBS and then incubated in block solution (10% serum of the animal the secondary antibody was raised in; 2% BSA; 0.1% Triton X100 in PBS) for 1 h at room temperature. The cells were incubated overnight with primary antibody (1/1000) in block solution and then at room temperature for 1 h in biotinylated horse anti-mouse secondary antibody in 1% serum (of the animal the secondary antibody was raised in), plus 2% BSA in PBS. After incubation with secondary antibody, the cells were washed three times in PBS for 5 min and then for 45 min in ABC (avidin-biotin-HRP conjugate) elite detection kit reagent (Vector Labs Inc.). Following three washes in PBS, the peroxidase was visualised by incubation in 3,3′-diaminobenzidine solution (Sigma) for 5 min. Controls included cells incubated either with no primary antibody, with pre-immune serum of the species used to raise the primary antibody, or with no secondary antibody.

## 5. Quantitative Real-Time PCR

The PCR reaction mix comprised 0.1 *μ*L cDNA, 0.2 *μ*M of each primer (see Table 1), and 1x QuantiTect SYBR Green master mix (Qiagen) in PCR grade H_2_O up to 20 *μ*L. The PCR program comprised a 15 min initial denaturation step at 95°C to activate the hot start polymerase, followed by 40 cycles of 95°C for 30 s, 55°C for 30 s, and 72°C for 30 s. The fluorescence signal was measured during the 72°C step of the cycle. Melt curve analyses to show generation of a single product for each reaction were carried out following the PCR program and comprised a slow temperature ramp from 60°C to 99°C, during which time the fluorescence signal was continuously monitored. Amplification of a single product of correct size was also confirmed by gel electrophoresis and verified by sequencing. Two to three independent analyses of duplicate reactions were undertaken for all samples. Data were analysed using the Rotor Gene 6 software (Corbett research) and initially converted into threshold values (*C*_*t*_), which refer to the cycle number during exponential amplification at which the PCR product crosses a set threshold. To adjust for variations in the amount of input cDNA, the average *C*_*t*_ values for the target gene were normalised against the average *C*_*t*_ values for GAPDH by the comparative quantitation method. 

## 6. Results

### 6.1. RhoA Gene Organisation Has Been Highly Conserved throughout Evolution and the Putative Promoter Contains Regulatory Elements Involved in Early Heart Development and Organogenesis

**Figure 1:**
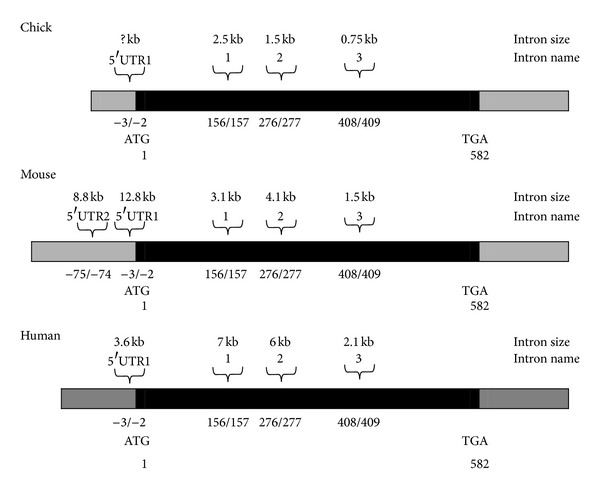
Comparative organisation of the chick, mouse, and human RhoA genes. Introns are indicated by their respective name and size above the cDNA line diagram and positioned as indicated by the nucleotide numbers below. Nucleotide position 1 refers to the A in the first in-frame ATG in the RhoA coding sequence. The open reading frame (ORF) is shown in black, with the start and stop codons and their respective nucleotide position shown below. The 5′ and 3′ untranslated regions (UTR) are depicted in grey.

We have previously shown that RhoA is necessary for normal heart formation in the developing chick [1]. In order to further investigate the regulation of RhoA expression in the early heart, the structure and organisation of the chick, mouse and human RhoA genes were obtained by genomic PCR analyses or comparative analysis of the known cDNA sequences against database genomic sequences. Subsequently, the putative promoter region of the mouse gene was deduced to permit the identification of *cis*-acting elements that might be involved in the regulation of expression of this gene in heart muscle. We first determined the genomic organisation of the chick RhoA gene by comparing the known cDNA sequence with sequence obtained from genomic PCR and sequence analysis (data not shown). Based on these analyses, the chick RhoA gene spans at least 7 kb and contains at least 4 introns. The first intron (designated Intron5′UTR1) is situated in the 5′UTR region at nucleotide position −3/−2 (i.e., upstream of the ATG translation start codon, where the “A” of the ATG is nt +1). This is presumably a very long intron (as is the case with both the mouse and human RhoA genes; see Figure 1); however, its length could not be determined by genomic PCR because the sequence of the 5′ UTR of chick mRNA is not known. In addition to this 5′UTR1 intron, three introns interrupt the chick RhoA open reading frame (ORF): Intron1 (2554 bp), situated between nucleotides (nts) 156 and 157; Intron2 (1549 bp), between nts 276 and 277; and Intron3 (750 bp), between nts 408 and 409 (see Figure 1). Introns1 and 2 display consensus splice site sequences consistent with the GT/AG rule [7], but the Intron3 5′-splice donor sequence is GC. Based on these results for the chick RhoA gene, the genomic organisation of the mouse and human RhoA genes was deduced by comparative analysis of the known cDNA sequences against sequences in the respective genomic sequence databases. Alignment of the deduced chick RhoA gene organisation to that of mouse and human RhoA genes reveals a high degree of conservation for both coding sequence (alignment of the chick and human RhoA cDNA sequences is shown in our earlier paper [1]) and gene organisation (is shown in Figure 1). The mouse and human RhoA genes contain introns situated at the same positions relative to the ORF as the four introns of the chick gene. The mouse RhoA gene contains an additional intron upstream of Intron5′UTR1, designated Intron5′UTR2. 

**Figure 2:**
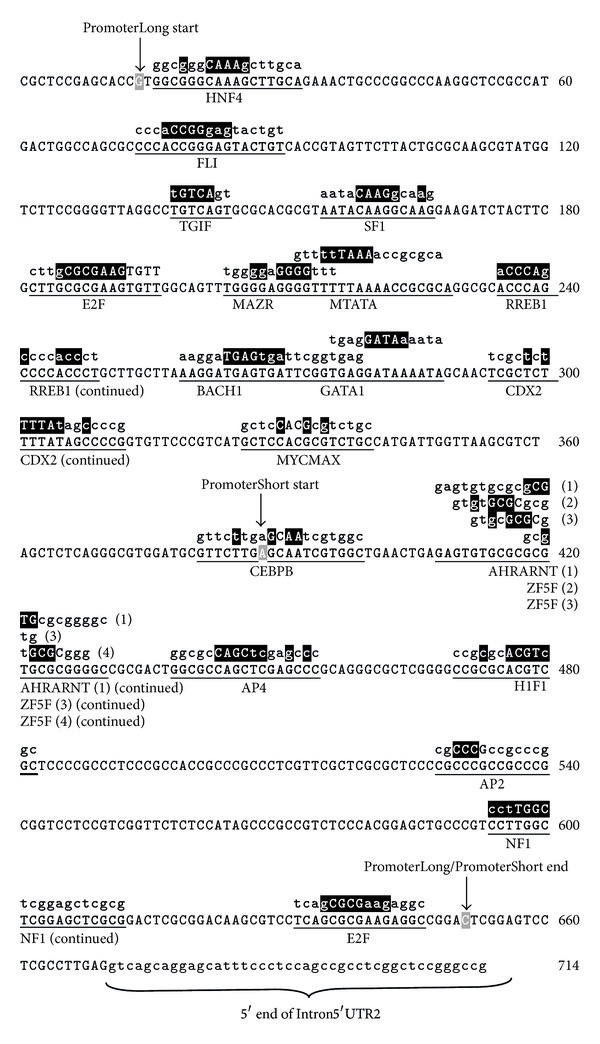
Putative mouse RhoA promoter and transcription factor binding sites. The promoter region of mouse RhoA was predicted using the Gene2 promoter program (http://www.genomatix.de). The first and last nucleotides for both regions identified are in grey-boxed white text and depicted by an arrow and respective name (start, end) above the sequence. Putative *cis*-acting transcription factor binding sites (identified by MatInspector with a core similarity of 1 and matrix similarity above 0.8) are labelled and underlined. The nucleotides boxed in black above the consensus sequence indicate that the matrix exhibits high sequence consensus at this particular position; the nucleotides in capital letters denote the core sequence. The nucleotide numbering shown in this figure is arbitrary—the “C” nt shown at the end of the PromoterLong/PromoterShort sequences is 5′ of Intron5′UTR2 and corresponds to nt −94 of the mouse RhoA cDNA, where the “A” of the start ATG is designated nt +1.

To provide initial information on the potential significance of this evolutionary conservation of the RhoA gene and encoded protein in terms of function in mouse cardiomyocyte differentiation, the promoter region of mouse RhoA was identified and analysed using a bioinformatics approach to identify relevant *cis*-acting promoter elements. Two different length fragments immediately upstream of mouse RhoA Intron5′UTR2 were identified as putative promoter regions using the Gene2 promoter program (http://www.genomatix.de): PromoterLong (640 bp in length) and PromoterShort (the most 3′ 230 bp of PromoterLong) (Figure 2). These regions were analysed for consensus transcription factor (TF) binding sites using MatInspector (http://www.genomatix.de). Eighteen putative TF binding sites were identified for the PromoterLong sequence and seven for PromoterShort (see Figure 2). 

As will be discussed later, many of the TF binding sites identified in these two different length regions are known to play an important role in early embryonic/heart development, implicating a role for RhoA in these processes. The abundance of transcription elements in these putative promoter regions is also consistent with these regions encompassing the true promoter. 

### 6.2. The Activity of the Putative RhoA Short Promoter Is Increased in P19CL6 Cells Induced to Differentiate into Cardiomyocytes

**Figure 3:**
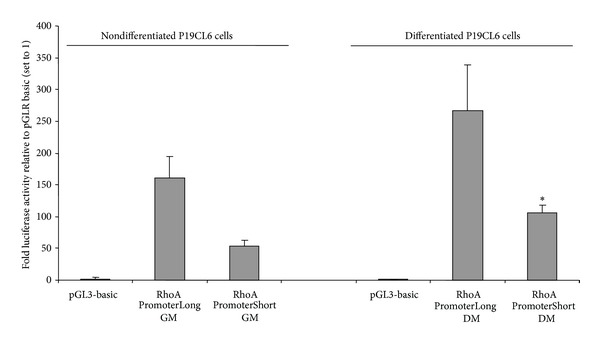
Relative promoter activity of PromoterLong and PromoterShort in nondifferentiated and cardiomyocyte-differentiated P19CL6 cells. Constructs containing different lengths of the putative promoter region of mouse RhoA (PromoterLong and PromoterShort) were cloned upstream of the firefly luciferase coding sequence in pGL3-Basic and cotransfected with the pRL-TK coreporter vector (expresses *Renilla* luciferase) into nondifferentiated and differentiated P19CL6 cells 11 days after DMSO induction. The firefly luciferase activity of each sample (expressed as relative light units) was normalised to the *Renilla* luciferase activity and expressed as this ratio (fold luciferase activity). The data represents the mean ± SEM of six individual transfections for each construct. *P < 0.05 compared to activity in nondifferentiated cells (Student's *t*-test).

To determine whether the two different length fragments of the putative promoter region of mouse RhoA exhibited promoter activity in differentiating heart cells, luciferase reporter assays were performed using mouse-derived P19CL6 cells, which form cardiomyocytes following growth for 10-11 days in a differentiation medium containing 1% DMSO [6]. Using this protocol, induced cells showed typical cardiomyocyte morphology, whereas noninduced P19CL6 cells (in growth medium lacking DMSO) showed no signs of differentiation (data not shown). Cells were subsequently cotransfected with pGL3-Basic vector containing the PromoterLong or PromoterShort putative promoter sequence cloned immediately upstream of a firefly luciferase reporter sequence and pRL-TK vector alone (containing the *Renilla* luciferase reporter, for normalising reporter expression). The cells were harvested 48 h later and promoter activity determined by assaying luminescence. The PromoterShort sequence showed more than 300-fold higher normalised luciferase activity in both noninduced and induced P19CL6 cells than the pGL3-Basic vector alone, indicating strong promoter activity; however, the PromoterLong sequence was 3-4 times more active again (Figure 3). The PromoterShort fragment is GC-rich (see Figure 2) and contains two core promoter elements, ZF2 and E2F [8], suggesting that this sequence encompasses the core promoter where orientation and initiation of transcription take place. The luciferase reporter assays indicate that elements upstream of this region within the PromoterLong region increase this core promoter activity, suggesting that this additional sequence encompasses the proximal promoter region. In terms of RhoA activity in differentiating versus nondifferentiating heart cells, it was observed that promoter activity for PromoterShort was significantly higher (*P* < 0.05) in differentiated P19CL6 than nondifferentiated P19CL6 cells. A similar change was observed for PromoterLong, but the results were not statistically significant. Nevertheless, these results overall support the hypothesis that RhoA plays an important role in the process of early cardiogenesis in the mouse. 

### 6.3. Inhibition of RhoA Blocks Differentiation of P19CL6 Cells into Cardiomyocytes

To indirectly assess the role of RhoA in differentiating mouse cardiomyocytes, we generated three P19CL6 cell lines stably expressing a dominant negative form of RhoA (mRhoAN19) and three cell lines that were mock (vector only) stably transfected. Incorporation of the vector (and RhoA construct sequence) into the genome of the different P19CL6 cell lines was confirmed by genomic PCR and sequencing (results not shown). Western blot analyses indicated that the RhoA levels in the three mock transfected cell lines, normalised to levels of *β*-actin, were similar to levels of the wild-type (wt) P19CL6 cell line (not shown). The levels of RhoA in two of the cell lines expressing the dominant negative form of RhoA (mRhoAN19 #2 and 3) were approximately 80% of wt levels, whereas RhoA levels for those the third clone (mRhoAN19 clone #1) were approximately 20% higher again than for the mock transfected cell lines; we inferred from these results that mRhoAN19 clone #1 exhibited the highest expression of the dominant negative form of RhoA.

**Figure 4:**
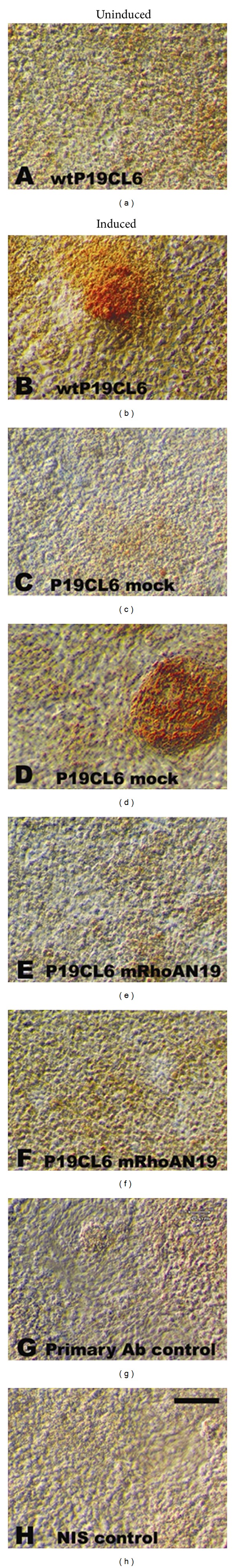
A dominant negative form of RhoA blocks differentiation of P19CL6 cells into a cardiomyocyte-like phenotype. P19CL6-derived cell lines were grown in growth medium or differentiation medium (growth medium containing 1% DMSO) in 24-well culture dishes from day of seeding and induction (D_0_) for 16 days (D_16_). Differentiated cardiomyocyte clusters (D_16_) were detected using a cardiac troponin-I (cTnI) antibody. (a)–(f) P19CL6 cell lines processed with cTnI antibody: wt P19CL6, wild-type (nontransfected) P19CL6 cells; P19CL6 mock, P19CL6 cells stably transfected with expression plasmid alone; P19CL6mRhoAN19, P19CL6 cells stably transfected with plasmid expressing dominant negative mouse RhoAN19; (g) primary antibody (Ab) control (i.e., lacking primary antibody); (h) nonimmune mouse serum (NIS) control. Addition of 1% DMSO resulted in differentiation into a cardiomyocyte-like phenotype in P19CL6 cells and the mock transfected P19CL6 cell lines but not in the dominant negative mutant (P19CL6mRhoAN19) cells. (b), (d) Beating clusters, once processed for cTnI immunodetection displayed cTnI-positive cell clusters (red/brown staining). Bar = 10 *µ*M.

To provide a measure of cardiomyocyte differentiation in these cells, we performed immunocytochemical analyses to qualitatively assess cardiac troponin-I (cTnI) levels and correlated these results with observation of phenotypic change. All cell lines were plated out in both growth medium (GM) and differentiation medium (DM: GM plus 1% DMSO) and grown for 16 days under identical conditions. Immunocytochemical detection of cTnI was carried out for each cell line (noninduced and induced with 1% DMSO) fixed at 8 different time points: 2, 4, 6, 8, 10, 12, 14, and 16 days after induction. We observed that the wt P19CL6 cells and the mock transfected P19CL6 cell lines (clones #1, 2, 3) exhibited a similar phenotype and maintained this phenotype over the growth period in GM; these cells did not differentiate (form beating clusters) and were cTnI-negative (see Figures 4(a) and 4(c)). Addition of 1% DMSO to both the P19CL6 cells and the mock transfected P19CL6 cell lines resulted in differentiation into a cardiomyocyte-like phenotype. Beating clusters, once processed for cTnI immunodetection displayed a temporal increase in the number and intensity of cTnI-positive cell clusters (red/brown staining, Figures 4(b) and 4(d)). cTnI staining was not detected in any of the negative controls: no primary antibody (Figure 4(g)); nonimmune mouse serum (Figure 4(h)). 

We next tested the effect of a dominant negative form of mRhoA on this established model system. In normal growth medium, all three cell lines stably transfected with the mRhoAN19 expression vector grew more slowly than both types of control cell line (wtP19CL6 and mock transfected cells), and after reaching confluency at day 4, their phenotype was stable over the 16-day growth period. cTnI staining indicated that these cells had not differentiated into a cardiomyocyte-like phenotype. However, these cell lines differed markedly in their response to DMSO-induced differentiation. Multiple independent experiments demonstrated that mRhoAN19 clone #1 cell line was unable to differentiate into a cardiomyocyte-like phenotype over 16 days: this was assessed as both lack of cell phenotypic change and absence of cTnI-positive cardiomyocyte clusters (Figure 4(f)). In contrast, mRhoAN19 clones #2 and #3 differentiated in a similar manner and timeframe to wt and P19CL6 mock transfected cells, as confirmed by the presence of cTnI-stained cardiomyocyte clusters (data not shown). We infer from these results that, in keeping with higher level of total RhoA measured, only cell line #1 expressed mRhoAN19 at a level sufficient to inhibit the activity of the endogenous RhoA and thereby block the process of differentiation into cardiomyocytes. These findings thus provide further strong evidence implicating RhoA as a necessary factor for cardiac differentiation. 

### 6.4. RhoA Inhibition Leads to an Accumulation of Cardiac Markers in Induced P19CL6 Cells

**Figure 5:**
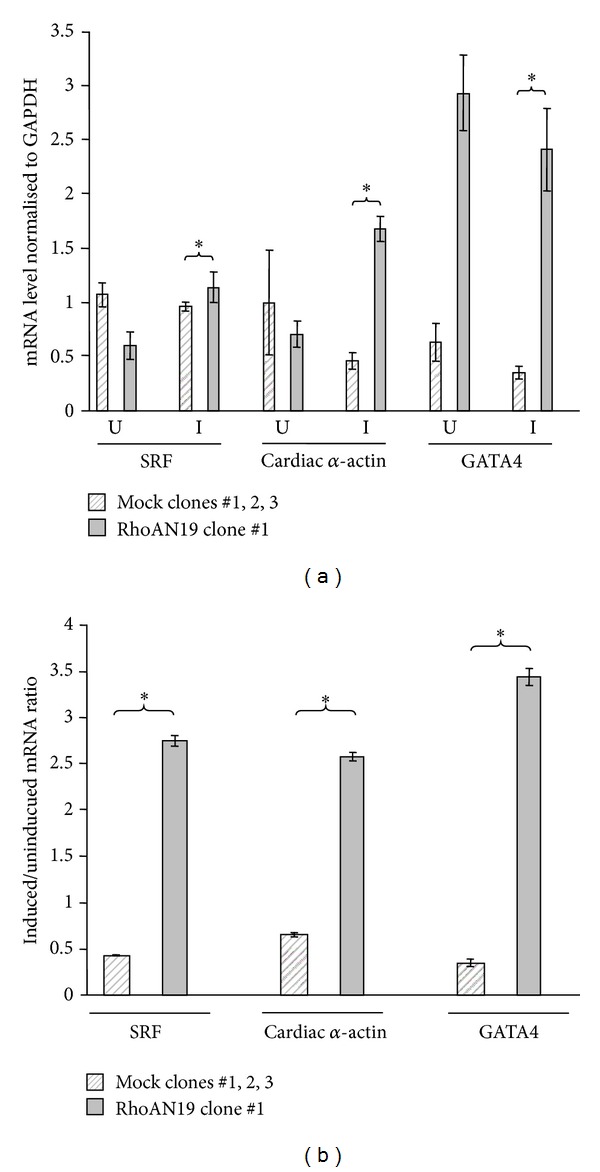
mRNA levels of selected genes involved in heart development in noninduced and induced P19CL6 cells. RNA was extracted from the different P19CL6 cell lines after growth in GM (uninduced, U) and DM (induced, I) for 14 days, then subjected to qPCR. The mRNA expression levels were normalised to GAPDH. Values for mock transfected (Mock) clones #1, 2, 3 represent combined results for these three cell lines. Values represent the mean ± SEM of three to six independent experiments. *P < 0.05, compared to mock transfected cell lines.

To test whether RhoA is involved in the transcriptional control of factors implicated in cardiomyocyte differentiation, mRNA levels of SRF, cardiac *α*-actin, and GATA4 in noninduced and induced P19CL6 cell lines were measured by quantitative real-time RT-PCR and normalised to GAPDH levels. For these analyses we compared the three mock transfected cell lines with mRhoAN19 clone #1 alone. We found that SRF, cardiac *α*-actin, and GATA4 mRNA levels for the noninduced P19CL6 RhoAN19 clone #1 were similar to those of the mock transfected cell lines (Table 2, column “U,” Figure 5). In contrast, there was a marked increase in SRF (3.7-fold), cardiac *α*-actin (4.7-fold), and GATA 4 (6.9-fold) mRNA levels in the induced P19CL6 RhoAN19 clone #1 cell line compared to the induced mock transfected clones (Table 2, column “I,” Figure 5(b)). Thus, the marked upregulation of these cardiac markers appears to parallel to the inhibition of cardiomyocyte differentiation seen in this cell line. Table 2 also summarises the ratios of the mRNA levels in the induced P19CL6 cell lines compared to noninduced P19CL6 cell lines (I/U). It should be noted that the mRNA levels of SRF, cardiac *α*-actin, and GATA4 were in general higher in noninduced cells than in induced cells (I/U ratio). Possible reasons for this observation are discussed later. In contrast, mRNA ratios (I/U) for SRF, cardiac *α*-actin, and GATA4 were markedly higher in the induced P19CL6 RhoAN19 clone #1 cell line. These results imply that inhibition of RhoA, in preventing the cardiomyocyte differentiation, resulted in a marked upregulation in expression of SRF, cardiac *α*-actin, and GATA4. 

## 7. Discussion

### 7.1. The RhoA Promoter Contains Regulatory Elements Implicated in Early Heart Development and Is Active in Differentiated Cardiomyocytes

In order to investigate the regulation of RhoA expression in early cardiogenesis, the mouse RhoA promoter was identified and isolated for functional analyses. Two different putative promoter fragments were tested and both showed high activity, with the PromoterLong fragment exhibiting three times more activity than the PromoterShort fragment. Not unexpectedly, a number of different consensus TF binding sequences were identified in this RhoA promoter, consistent with the various known functions of the RhoA protein in different tissue types and/or at different developmental stages. Importantly, many of the potential TF binding sites identified have been reported to be involved in either early heart development and/or early embryogenesis. The 5′ end of the PromoterLong fragment contains the FLI (ETS family member FLI), TG1F (TG-interacting factor belonging to TALE class of homeodomain factors), and E2F transcription factor sites. FLI has been implicated in normal head and heart development, as well as in erythroid differentiation, while TG1F has been shown to be a coactivator of embryonic development [9–11]. E2F is known to be involved in cell cycle regulation by interaction with Rb p107 protein factor in a proliferation-dependent signal transduction pathway, and is also implicated in control of cardiac myocyte growth [12, 13]. The 3′ end of the PromoterLong fragment, encompassing the PromoterShort fragment, contains core promoter elements and is particularly rich in consensus binding sites for transcription factors implicated in early heart development and/or embryogenesis. The AHR-ARNT (aryl hydrocarbon receptor/AhR nuclear translocator) heterodimers are coexpressed in the developing chick heart and potentially have role in cardiac development and growth [14, 15]. The AP4 (activator protein 4) is possibly involved in the regulation of the *β*-myosin heavy chain gene in rat cardiomyocytes, and H1F-1 (hypoxia induced factor 1 (HIF-1)) has been shown to be essential for normal cardiac development [16, 17]. AP2 (activator protein 2) is developmentally regulated, and cardiac neural crest cells expressing AP2 have been shown to be important for cardiac looping in zebrafish [12, 18]. The NF1 (nuclear factor 1) is an important regulator in the RAS signal transduction pathway and for normal embryogenesis and normal heart development [19, 20]. As mentioned above, E2F has been shown to be implicated in the control of cardiac myocyte growth and is a core promoter element [8]. The two core promoter elements, ZF5 (found in 50.75% of human core promoters) and E2F (found in 74.25% of human core promoters) [8], are represented in the PromoterShort fragment. In addition, this region is GC-rich, which is common for core promoters [21]. These combined features suggest that the PromoterShort fragment contains or is the core promoter of RhoA. This conclusion is also supported by the results obtained from the luciferase reporter assays, which indicate that PromoterShort has less promoter activity than PromoterLong. The increased promoter activity for PromoterLong indicates that elements upstream of PromoterShort are necessary to enhance the promoter activity, suggesting that this region is the proximal promoter of RhoA. The increased activity of the RhoA PromoterShort fragment in differentiating cardiomyocytes compared to nondifferentiated cells indicates that RhoA has an important role in differentiating cardiomyocytes and suggests that some of the transcription factors expressed in differentiating P19CL6 cells bind to the RhoA promoter to increase transcriptional activity. These results support earlier findings, which implicate RhoA as an important factor in early heart development and normal embryogenesis. 

The putative human RhoA promoter was previously cloned to investigate the PKG-(cGMP-dependent-protein kinase-) dependent regulation of RhoA in arterial smooth muscle cells [22]. Two different length fragments (913 bp and 118 bp) just upstream of the ATG codon were assayed for luciferase activity, and, interestingly, the shorter fragment exhibited the same promoter activity as the longer fragment. With the results reported here, these data suggest that the activity of the RhoA promoter is dependent on tissue type and developmental stage.

### 7.2. Inhibition of RhoA Blocks Differentiation of P19CL6 Cells into Cardiomyocytes and Causes an Accumulation of Cardiac Markers

Clonal P19CL6 cell lines expressing a dominant negative mutated form of RhoA (RhoAN19) were generated to investigate the potential role of RhoA in differentiating cardiomyocytes. Phenotypic observation and cTnI staining of one clone (P19CL6 mRhoAN19 clone #1) showed that these cells were unable to differentiate into cardiomyocytes upon induction with 1% DMSO. This result implicates RhoA as an important factor for the differentiation of cardiomyocytes. In addition, our observations suggest that factors other than RhoA are responsible for the control of the cytoskeleton and cell adhesion of P19CL6 cells, as the P19CL6 mRhoAN19 clone #1 cells grew well, attached as normal, and had the same apparent morphology as noninduced wt P19CL6 and P19CL6 mock transfected cells. In keeping with this conclusion earlier findings demonstrate that inhibition of all the Rho GTPases in cardiac muscle cells does not disrupt actin muscle fiber morphology [23]. Together, then, these results suggest that actin morphology is not regulated by RhoA-dependent pathways in cardiac precursor cells and cardiac muscle cells.

In order to investigate whether RhoA is involved in the transcriptional regulation of targets known to be involved in early heart development, real-time quantitative PCR measurements of specific target gene mRNA levels were performed. Interestingly, SRF, cardiac *α*-actin, and GATA4 were all markedly upregulated in the DMSO-treated, differentiation-blocked P19CL6 RhoAN19 clone #1 cell line but not in any of the other cell lines. This effect of RhoA inhibition is in accordance with earlier studies demonstrating that inhibition of RhoA associated kinases, direct down-stream effectors of RhoA, leads to increased expression of SRF, cardiac *α*-actin, and GATA4 in stage 8 chick embryos [4]. Thus, these earlier results and our findings indicate that inhibition of RhoA not only prevents cardiomyocyte differentiation but also causes an accumulation of these cardiac markers.

The real-time analyses from this study also indicated that the mRNA levels of SRF, cardiac *α*-actin, and GATA4 were higher in noninduced than DMSO-induced P19CL6 cells grown for 14 days. P19CL6 cells are putative cardiac precursor cells and it is formally possible that these cells, when grown under specific conditions of confluency, as in these experiments as a prelude to DMSO treatment, are driven, at least partially, in the direction of cardiomyogenesis. In the absence of DMSO, however, the cells would lack the trigger necessary to move them into the differentiation phase. In contrast, once having differentiated into cardiomyocytes, a process dependent on functional RhoA, these early heart-specific factors might no longer be required to the same extent, with their expression subsequently downregulated. This premise is compatible with another finding in this study, namely the accumulation of cardiac markers in P19CL6 RhoAN19 clone #1 cell line, in which DMSO-induced cardiomyocyte differentiation was blocked. In this case, we propose that the factor(s) induced by DMSO is not activated due to the abrogation of RhoA activity such that the cells are blocked at a predifferentiation stage in which factors necessary for the differentiation process have accumulated. The corollary of this argument is that under normal conditions, RhoA signals cardiac differentiation and releases the accumulation of cardiac transcription factors.

Normally, P19CL6 cells differentiate into cardiomyocytes when induced with DMSO. Inhibition of RhoA prevented this differentiation. The results from this study suggest that the factors induced by DMSO are involved in a RhoA-dependent signalling pathway. Whether RhoA is downstream of this unknown factor or upstream and activates the factor through binding, either directly or indirectly, remains unknown. It is also possible that RhoA activates transcription of these unknown factors. These are important future questions that will need to be investigated in order to understand the processes of cardiomyocyte differentiation at the molecular level. 

## Figures and Tables

**Table 1:** Primers used for PCR.

PCR product	Forward primer (5′-3′)	Reverse primer (5′-3′)	Product size (bp)
Elucidation of chick RhoA gene structure			

Chick RhoA Intron1	GCCGGAATTCATGGCAGCCATTCGAAA^1^	CTATCAGGACTATCGATTG	282 bp^4^
Chick RhoA Intron2	GTGGAGTTGGCTTTGTGGGATAC	GCTTCATTTTGGCCAGCTCT	251 bp^4^
Chick RhoA Intron3	AGTGGACCCCGGAAGTGA	TAAGAGAAGGCACCCGGAC	287 bp^4^

Amplification of putative mouse RhoA promoter			

PromoterLong	CCCGCGGATCCGTGGCGGGCAAAGCTTGCAG^2^	CCCGCGGATCCGTCCGGCCTCTTCGCGCT	637
PromoterShort	CCCGCGGATCCAGCAATCGTGGCTGAACTGAG^2^	CCCGCGGATCCGTCCGGCCTCTTCGCGCT	264

Generation of dominant mutant of mouse RhoA			

mRhoA N19	CGCCGCTCGAG**ATG**GCTGCCATCAGGAAGAAACTGGTG**ATT**GTT GGTGATGGAGCTTGTGGTAAGAATTGCTTGCTCATA^3^	ACGCGTCGACTCACAAGATGAGGCACCC	582

Primers used for real-time PCR			

GAPDH	TCCTACCCCCAATGTGTCCGTC	GCCCAAGATGCCCTTCAGTG	121
RhoA ORF	ATTGATGTGTTTTTCCATTG	CTCCCGTCTCGTGTGCTCGTCATT	151
RhoA 3′UTR	GCTACCAGTATTTAGAAGCCAACCAC	GCTGTTAGAGCAGTGTCAGAAGGAC	88
SRF	TGCCTCAACTCGCCAGACTCTC	TTCAGTGTGTCCTTGGTTTCCC	140
Cardiac *α*-actin	GCCAACCGTGAGAAGATGACC	CGCCAGAATCCAGAACAATGC	130
GATA 4	ATGCCGAGGGTGAGCCTGTATG	CTTCCGTTTTCTGGTTTGAATCC	110

^1^Incorporated* EcoR1* and ^2^ *BamH1* sites are underlined.

^3^ATG translation start codon and introduced N19 mutation are shown in bold.

^4^Expected size in *absence* of intron.

**Table 2:** mRNA levels of selected genes involved in heart development in P19CL6 cell lines with and without induction to generate a cardiomyocyte phenotype^1^.

Gene	Cell line^2^	mRNA level: noninduced cells (U)	mRNA level: induced cells (I)	I/U^3^
SRF	Mock clones #1, 2, 3	1.08 ± 0.11	0.46 ± 0.08	0.43 ± 0.01
	mRhoAN19 clone #1	0.60 ± 0.12	1.68 ± 0.11*	2.75 ± 0.06*
Cardiac *α*-actin	Mock clones #1, 2, 3	0.97 ± 0.04	0.63 ± 0.17	0.65 ± 0.02
	mRhoAN19 clone #1	1.14 ± 0.14	2.93 ± 0.35*	2.57 ± 0.04*
GATA4	Mock clones #1, 2, 3	1.00 ± 0.48	0.35 ± 0.05	0.35 ± 0.04
	mRhoAN19 clone #1	0.70 ± 0.12	2.41 ± 0.38*	3.44 ± 0.09*

^1^Data represent the results of 3–6 independent measurements.

^2^Values for mock transfected (Mock) clones #1, 2, 3 represent combined results for these three cell lines.

^3^I/U is the ratio of mRNA levels in induced (I) and uninduced (U) cells.

*P < 0.05, compared to mock transfected cell lines.
